# Prognostic significance of stem cell/ epithelial-mesenchymal transition markers in periampullary/pancreatic cancers: FGFR1 is a promising prognostic marker

**DOI:** 10.1186/s12885-020-6673-2

**Published:** 2020-03-14

**Authors:** Yosep Chong, Nishant Thakur, Kwang Yeol Paik, Eun Jung Lee, Chang Suk Kang

**Affiliations:** 1 Department of Hospital Pathology, Yeouido St. Mary’s Hospital, College of Medicine, The Catholic University of Korea, 10, 63-ro, Yeongdeungpo-gu, Seoul, 07345 Republic of Korea; 2 Department of Surgery, College of Medicine, The Catholic University of Korea, Seoul, 07345 Republic of Korea; 3 Department of Pathology, Shinwon Medical Foundation, Soha-ro 109 beon-gil, Gwanmyeong-si, 14316 Gyeonggi-do Republic of Korea; 4 Department of Pathology, Samkwang Medical Laboratories, 57, Baumoe-ro 41-gil, Seocho-gu, Seoul, 06742 Republic of Korea

**Keywords:** Pancreatic ductal carcinoma, Duodenal neoplasms, Common bile duct neoplasm, SOX transcription factor, Duodenal neoplasms, Fibroblast growth factor receptor, Octamer transcription factor-4

## Abstract

**Background:**

Periampullary cancers (PAC) including pancreatic, ampulla of Vater (AOV), and common bile duct (CBD) cancers are highly aggressive with a lack of useful prognostic markers beyond T stage. However, T staging can be biased due to the anatomic complexity of this region. Recently, several markers related to cancer stem cells and epithelial-mesenchymal transition (EMT) such as octamer transcription factor-4 (Oct4) and fibroblast growth factor receptor 1 (FGFR1) respectively, have been proposed as new promising markers in other solid cancers. The aim of this study was to assess the expression and prognostic significance of stem cell/EMT markers in PACs.

**Methods:**

Formalin-fixed, paraffin-embedded tissues of surgically excised PACs from the laboratory archives from 1998 to 2014 were evaluated by immunohistochemical staining for stem cell/EMT markers using tissue microarray. The clinicopathologic parameters were documented and statistically analyzed with the immunohistochemical findings. Survival and recurrence data were collected and analyzed.

**Results:**

A total of 126 PAC cases were evaluated. The average age was 63 years, with 76 male and 50 female patient samples. Age less than 74 years, AOV cancers, lower T & N stage, lower tumor size, no lymphatic, vascular, perineural invasion and histologic well differentiation, intestinal type, no fibrosis, severe inflammation were significantly associated with the better overall survival High expression levels of FGFR1 as well as CK20, CDX2, and VEGF were significantly related to better overall survival, while other stem cell markers were not related. Similar findings were observed for tumor recurrence using disease-free survival.

**Conclusions:**

In addition to other clinicopathologic parameters, severe fibrosis was related to frequent tumor recurrence, and high FGFR1 expression was associated with better overall survival. Histologic changes such as extensive fibrosis need to be investigated further in relation to EMT of PACs.

## Background

Periampullary cancers (PAC) including pancreatic, ampulla of Vater (AOV), and common bile duct (CBD) cancers are highly aggressive tumors, and their 5-year survival rate is less than 4, and 90% of the patients die from the disease within a year after diagnosis [1]. PACs are expected to rank as the second leading cause of cancer death in 10 years after lung cancers [2]. This is presumed to be because of the invasive growth of the tumor, delayed diagnosis due to the absence of specific symptoms, and limited treatment options. To date, the T stage, which represents the operability of curative resection is the most convincing prognostic marker, according to the American Joint Cancer Committee/Union International Contre le Cancer staging system (AJCC/UICC) [1]. However, T stage evaluation completely depends on the pathologist’s decision based only on gross and pathologic examination, which can be subjective by individuals due to the anatomical complexity of this region [2].

Moreover, as the tumor grows and invades more than two adjacent tissues, such as duodenum and AOV, or distal CBD and pancreatic head, determining the epicenter of the tumor is not straightforward, and might not produce reproducible results between examiners. In a recent review by Adsay et al., the T stage in 39% of the examined cases differed from the original reports [2]. Moreover, the T staging system is different for each cancer, which depends on the primary location (Supplementary Table 1) [3]. For example, a tumor that lies on the distal CBD and AOV with duodenal involvement is T3 for CBD cancer while it is T2 for AOV cancer according to the AJCC cancer staging 7th edition; There’s a higher chance to get the same results by 8th edition that the CBD tumors with duodenal involvement would likely invade the bile duct wall with a depth greater than 12 mm. According to the official guidelines of AJCC, the survival of patients with T1 and T2 stages was reversed during the first year after surgery [3]. Therefore, there is an immediate need for a feasible substitute for proper prognostic anticipation, but thus far, no effective markers have been developed.

Recent studies have found that many solid tumors, such as breast and colon cancers, are composed of heterogeneous tumor clusters showing different molecular genetic characteristics. Cancer stem cells are believed to play a major role in tumor development, progression, metastasis, and resistance. More recently, in pancreatic cancer cell lines, stem cell markers, such as Oct4, NANOG, and SOX2, were found to be aberrantly overexpressed compared to normal pancreatic cell lines [4–6]. This aberrant overexpression resulted in uncontrolled proliferation and dedifferentiation of tumor cells by causing changes in the expression of genes that control the G1/S phase of the cell cycle, and epithelial-mesenchymal transition (EMT) [4, 7–9]. Likewise, several EMT markers such as FGFR1, IGF-1, VEGF, and recently ZEB1/2, SNAIL/SLUG, are being considered as promising prognostic markers [10, 11]. Moreover, these markers are thought to be very important as potential targets for tailored therapy. However, the expression of these markers and their prognostic significance in clinical cases of PACs have not been fully studied.

The purpose of this study was to assess the expression and prognostic significance of the stem cell markers and the markers related to EMT in PACs, and finally to identify a panel of prognostic markers that can classify PACs into more relevant groups.

## Methods

### Patients and samples

This study was approved by the Institutional Review Board of the Catholic University of Korea (SC14SISI0052). We investigated formalin-fixed, paraffin-embedded tissues of surgically excised PACs from our laboratory archives (Yeouido St. Mary’s Hospital, Seoul, Korea) from 1998 to 2014 (15 years). These included pancreatic head cancers, distal CBD cancers, AOV cancers, and duodenal cancers with periampullary involvement, excised by Whipple surgery or pylorus-preserving pancreaticoduodenectomy. None of the cases had undergone any type of preoperative chemotherapy. We sequentially retrieved a total of 126 cases after excluding intraductal papillary mucinous neoplasms, which are considered as benign or premalignant lesions, metastatic cancers from other organs, and duodenal cancers without ampullary or pancreatic involvement. The mean age of the enrolled patient cases was 63 years (ranging from 36 to 82 years), and out of 126 cases, 76 were male, and 50 were female. The retrieved cases were blinded by sequential numbering. The hematoxylin and eosin-stained slides were independently reviewed by two pathologists (Y. Chong and EJ. Lee) to confirm the original diagnosis. The cases with not enough tissue available for tissue microarray analysis were excluded. The epicenter of the tumors was reevaluated and compared with the evaluation from the original diagnosis.

The clinicopathologic data were documented: location of the mass, gross type (fungating/polypoid, sessile, ulceroinfiltrative), presence of ulceration, tumor size (largest diameter), radial resection margin involvement, TNM stage, lymphatic, vascular, perineural invasion, histologic differentiation (pancreaticobiliary vs. intestinal subtype, and well, moderately, poorly for each type), degree of fibrosis and inflammatory cell infiltration (mild, moderate, severe), date of surgery, follow-up duration, date of recurrence, and date of death. The degree of fibrosis was described by the fourth -tier system based on H&E finding (none (< 10%), mild (10–33%), moderate(34–66%), and severe (67–100%)). The epicenter of the tumor was reevaluated for each case and compared with the original diagnosis, based on the staging system of AJCC/UICC 7th edition because most of the cases were originally diagnosed based on the 7th edition. T stage was rescored according to the revised tumor epicenter and compared with the original T stage. Pancreaticobiliary and intestinal subtype was determined based on the results of immunohistochemical staining for CK7, CK20, CDX-2, and MUC-2 (Mucin 2),. Information regarding the cause and date of death was collected based on National Death Certificate data and medical records from our institute. Since National Death Certificate records have a year of delay for data collection, the death data of recent cases of 2013 and 2014 were documented according to our institutional medical records. Tumor recurrence was defined as a newly detected tumor or metastasis upon radiological examination such as computed tomography (CT) or position emission tomography (PET-CT) with or without an increase of serum CA19–9 level in the surgically resectable cases. Incompletely resected cases with progressive disease were excluded from assessment for tumor recurrence and, death resulting from early complications such as bleeding, bile leakage, infection, and pulmonary embolism were not considered as cancer-related death. Cases with the unknown or unspecified cause of death were excluded from survival analysis.

### Tissue microarray

Nine TMA recipient blocks were made using Quick-Ray® Tissue Microarray recipient block (UB06–2, UNITMA Co., LTD., Seoul, Korea). Three 2 mm sized tumor spots representing each case were taken from donor blocks to avoid tissue loss and edge artifact. Each recipient block consisted of 45 cores of tumor tissue (15 cases), and 4 cores of positive controls for stem cell markers, and 1 core of negative control. To reduce tissue loss, the recipient blocks were incubated at 30 °C for 25 min before core insertion. The positive control cores were normal lung alveoli and squamous cell carcinoma of the lung for CD24 and SOX2, testicular seminoma for Oct4, benign urothelial epithelium of the bladder for CD44v6, normal umbilical cord for FGFR1, normal blood vessel for VEGF, and normal liver tissue for IGF-1.

### Immunohistochemistry

The TMA blocks were cut into 4 μm thick sections, mounted on silanized glass slides, and dried in an oven at 70 °C for 60 min. The slides were automatically processed and stained by BenchMark XT (Ventana, Roche Diagnostics, USA) according to the manufacturer’s instructions. The procedure included antigen retrieval by heating at 70 °C for 1 h, followed by pretreatment with cell conditioner 2 (pH 6) for 60 min, and subsequent incubation with each antibody at optimal temperature for 32 min, and then counterstained by hematoxylin for 4 min and bluing agent for 4 min, followed by chromogenic detection using UltraView Universal DAB Detection Kit for mins, and final washing step for mins. The stained slides were covered with balsamic acid. Immunohistochemical staining was performed for CK7, CK20, CDX-2, and MUC-2 and the tumors were classified into pancreaticobiliary and intestinal subtype. In addition, immunohistochemical staining was performed for stem cell markers including CD24, SOX2, Oct4, and CD44v6 and epithelial-mesenchymal transition markers, including VEGF, IGF-1, and FGFR1. The dilution and incubation conditions for each antibody are as follows: CK7 (Prediluted, Ventana, Roche Diagnostics, USA), CK20 (Prediluted, Ventana, Roche Diagnostics, USA), CDX-2 (Prediluted, Ventana, Roche Diagnostics, USA), MUC-2 (Prediluted, Ventana, Roche Diagnostics, USA), CD24 (1:50, Thermo Scientific, UK), Oct4 (ChIP Grade ab19857, 1:100, Abcam, CB4, UK), SOX2 (SP76, 1:100, Cell Marque, CA, USA), VEGF (1:50, Quartett Immunodiagnostika, Berlin, Germany), IGF-1 (1:100, Abcam, CB4, UK), FGFR1 (ChIP Grade ab10646, 1:100, Abcam, CB4, UK) and CD44v6 (1:200, Invitrogen, CA, USA). The immunoreactivity was scored for each tissue microarray core as a three-tier system (0, 1+, 2+, 3+) according to the intensity. If the intensity is heterogeneously high or low, the most commonly found intensity was scored.

### Statistical analysis

All analyses were performed using R statistical software (version 3.2.3 via http://web-r.org/) and IBM SPSS Statistics (ver. 1.0.0.1347 64-bit). Statistical analysis was performed using the Mann-Whitney test for continuous variables and Fisher’s exact test or chi-square tests for categorical variables to compare the groups with and without recurrence For multivariable regression analysis, logistic regression analysis was used to evaluate the association between the clinicopathologic parameters and the recurrence.

Kaplan-Meier analysis was used for survival analysis to search the parameters affecting the overall survival and disease-free survival and results with a *p*-value less than 0.05 were considered as statistically significant. To find the novel panel of prognostic markers, the combined immunoreactivity score was made using the immunoreactivity of certain 2, 3, 4, 5 IHC markers and analyzed with overall survival. The group was divided into the lower and higher expression groups according to the expression level of the IHC panel. Using the statistically significant parameters in Kaplan-Meier analysis, Cox regression analysis was used to confirm the relationship and to determine the odds ratio of each parameter.

## Results

### Patient and tumor characteristics

The clinicopathologic data of the evaluated cases are summarized in Table 1. Mean age of the patients was 63 years (ranging from 36 to 82). There were 76 male patient cases and 50 female patient cases (M:F = 1.52:1). Originally, the epicenter of the tumor was diagnosed as periampullary duodenum in 3 cases, AOV in 37 cases, pancreatic head in 37 cases, distal CBD in 47 cases, and proximal CBD in 2 cases. After pathological revision, the epicenter of the tumor was AOV in 34 cases, pancreatic head in 44 cases, and distal CBD in 48 cases (Table 1). Changes in the original and revised diagnoses have been described in the following section and summarized in Table 2.

**Table 1:** Summary of clinicopathological data of the enrolled cases

	No. (%)			
Age (yrs)	Ranged 36 ~ 82		Mean 63.0 ± 9.4	
Sex	Male 76, Female 50		M:F = 1.52:1	
Location (tumor epicenter)	Original diagnosis		Revised diagnosis	
Periampullary duodenum	3	(2.4%)	0	(0%)
AOV	37	(29.4%)	34	(27.0%)
Pancreatic head	37	(29.4%)	44	(34.9%)
Distal CBD	47	(37.3%)	48	(38.1%)
Proximal CBD	2	(1.6%)	0	(0%)
T stage	Original diagnosis		Revised diagnosis	
Tis	1	(0.8%)	1	(0.8%)
T1	21	(16.7%)	21	(16.7%)
T2	29	(23.0%)	29	(23.0%)
T3	67	(53.2%)	67	(53.2%)
T4	8	(6.3%)	8	(6.3%)
Gross type				
Fungating	20		(15.9%)	
Infiltrative	93		(73.8%)	
Ulcerofungating	3		(2.4%)	
Sessile	5		(4.0%)	
Solid	2		(1.6%)	
Tumor size	Ranged 0.6 ~ 8.0 cm		Mean 3.2 ± 1.6 cm	
< 4.5 cm	96		(76.2%)	
≥ 4.5 cm	30		(23.8%)	
N stage				
N0	73		(57.9%)	
N1	53		(42.1%)	
N2	0		(0%)	
M stage				
M0	117		(93.7%)	
M1	8		(6.3%)	
	Absent		Present	
Lymphatic invasion	70	(55.6%)	56	(44.4%)
Vascular invasion	110	(87.3%)	16	(12.7%)
Perineural invasion	54	(42.9%)	72	(57.1%)
Positive radial resected margin	118	(93.7%)	8	(6.3%)
Tumor ulceration	116	(92.1%)	10	(7.9%)
Histologic grade	Original diagnosis		Revised diagnosis	
Well differentiated	37	(29.4%)	32	(25.4%)
Moderately differentiated	79	(62.7%)	90	(71.4%)
Poorly differentiated	10	(7.9%)	4	(3.2%)
Histologic subtype				
Pancreaticobiliary subtype	34		(27.2%)	
Prone to pancreaticobiliary subtype	58		(46.4%)	
Prone to intestinal subtype	19		(15.2%)	
Intestinal subtype	14		(11.2%)	
Degree of fibrosis	Original diagnosis		Revised diagnosis	
Absent	4	(3.2%)	1	(0.8%)
Mild	20	(15.9%)	35	(27.8%)
Moderate	72	(57.1%)	57	(45.2%)
Severe	30	(23.8%)	33	(26.2%)
Degree of inflammation	Original diagnosis		Revised diagnosis	
Mild	53	(42.1%)	42	(33.3%)
Moderate	62	(49.2%)	70	(55.6%)
Severe	11	(8.7%)	14	(11.1%)
Tumor recurrence	Absent		Present	
	64	(51.2%)	61	(48.8%)
Death	39	(31.0%)	87	(69.0%)
Follow-up duration (days)	Ranged 3 ~ 5234		969.7 ± 1135	
Disease free survival (days)	Ranged 3 ~ 4173		731.2 ± 954.5

**Table 2:** Comparison of tumor epicenter and T stages between original and revised diagnoses

(A)						
	Revised tumor epicenter					
Original tumor epicenter	Periampullary duodenum	AOV	Pancreatic head	Distal CBD	Proximal CBD	Total
Periampullary duodenum	0	2	0	1	0	3
AOV	0	29	3	5	0	37
Pancreatic head	0	2	33	2	0	37
Distal CBD	0	1	8	38	0	47
Proximal CBD	0	0	0	2	0	2
Total	0	34	44	48	0	126
(B)						
	Revised T stage					
Original T stage	Tis	T1	T2	T3	T4	Total
Tis	1	0	0	0	0	1
T1	0	21	0	0	0	21
T2	0	0	27	2	0	29
T3	0	0	2	64	1	67
T4	0	0	0	1	7	8
Total	1	21	29	67	8	126

Grossly, 20 cases (15.9%) were categorized as fungating type, 93 (73.8%) as infiltrative, 3 (2.4%) as ulcerofungating, 5 (4.0%) as sessile, and 2 (1.6%) as solid type. The mean tumor size was 3.2 cm (ranging from 0.6 to 8.0 cm). Since tumor size (> 4.5 cm) is one of the important prognostic markers for pancreatic cancer, the cases were divided into two groups based on tumor size (< or > than 4.5 cm) and compared. There were 96 cases with smaller tumor size (76.2%) and 30 cases with larger tumor size (23.8%). Tumor classification based on N stage according to the AJCC staging system, showed that 73 cases (57.9%) were N0, 53 were N1 (42.1%), and there were no N2 cases. M stage classification indicated that 8 cases (6.3%) were M1, while the rest was M0 (117 cases, 93.7%). Lymphatic invasion was found in 56 cases (44.4%), vascular invasion in 16 (12.7%), and perineural invasion in 72 cases (57.1%). Positive radial resection margin was found in 8 cases (6.3%). Tumor ulcer was found in 10 cases (7.9%).

Classification according to histologic grade showed that originally, 37 cases (29.4%) were diagnosed as well-differentiated tumors, 79 (62.7%) as moderately differentiated, and 10 as (7.9%) poorly differentiated. After review, 32 (25.4%) were well-differentiated, 90 (71.4%) were moderately differentiated, and 4 (3.2%) were poorly differentiated. In histologic subtypes, 34 cases (27.2%) were pancreaticobiliary subtype, 58 (46.4%) were more likely to be pancreaticobiliary subtype, 19 cases (15.2%) were more likely to be intestinal subtype, and 14 cases (11.1%) were of the intestinal subtype. Degree of accompanying fibrosis, known as desmoplastic reaction, was absent in 4 cases (3.2%) in the original pathologic reports, mild in 20 cases (15.9%), moderate in 72 cases (57.1%), and severe in 30 cases (23.8%). After revision, it was absent in 1 case (0.8%), mild in 35 cases (27.8%), moderate in 57 cases (45.2%), and severe in 33 cases (26.2%). Degree of peritumoral inflammation was mild in 53 cases (42.1%) in the original pathologic reports, moderate in 62 cases (49.2%) and severe in 11 cases (8.7%). After revision, it was mild in 42 cases (33.3%), moderate in 70 cases (55.6%), and severe in 14 cases (11.1%). Tumor recurrence was observed in 61 cases (48.8%) during an average follow-up of 969.7 days (ranged 3 to 5234 days). Disease-free survival duration was an average of 731.2 days (ranging from 3 to 4173 days). Eighty-seven out of 126 patients (69.0%) were dead during the follow-up.

### Comparison of tumor epicenter and T stages between original and revised diagnoses

Comparison of original and revised diagnoses showed that the epicenter of the tumor was altered in 22 out of 126 cases (17.4%) (Table 2). Among the 22 cases, 10 cases showed a discrepancy between distal CBD and pancreatic head cancers, 6 cases showed a discrepancy between distal CBD and AOV cancers, 5 cases showed a discrepancy between pancreatic head and AOV cancers, 2 cases showed a discrepancy between periampullary duodenum and AOV, 2 cases showed a discrepancy between proximal and distal CBD, and 1 case showed a discrepancy between periampullary duodenum and distal CBD. As the tumor locations have been changed after the review, the T stages were also altered (Table 2). As a result, 6 cases showed different T stage between the original and revised diagnoses with 3 overstaged and 3 understaged cases, respectively (6 out of 126 cases, 4.8%).

### Immunohistochemical staining and immunoreactivity results

The immunohistochemical staining conditions are summarized in Table 3 and the representative images of the immunohistochemical stainings are shown in Supplementary Fig. 1. The immunoreactivity of SOX2 in the nucleus was negative in 13 cases (10.3%), 1+ in 66 cases (52.4%), 2+ in 45 cases (35.7%) and 3+ in 2 cases (1.6%). The SOX2 immunoreactivity in the cytoplasm was negative in 66 cases (52.4%), 1+ in 56 cases (44.4%), 2+ in 4 cases (3.2%), and no cases showed 3+. CD24 staining was negative in 6 cases (4.8%), 1+ in 79 cases (62.7%), 2+ in 37 cases (29.4%), and 3+ in 4 cases (3.2%). Oct4 immunoreactivity was 1+ in 26 cases (20.6%), 2+ in 73 cases (57.9%), and 3+ in 27 cases (21.4%). IGF-1 staining was negative in 13 cases (10.3%), 1+ in 74 cases (58.7%), 2+ in 33 cases (26.2%), and 3+ in 6 cases (4.8%). The FGFR1 immunoreactivity was 1+ in 7 cases (5.6%), 2+ in 52 cases (41.3%), and 3+ in 67 cases (53.2%). The VEGF immunoreactivity was 1+ in 18 cases (14.6%), 2+ in 78 cases (63.4%), and 3+ in 27 cases (22.0%). CD44v6 staining was negative in 14 cases (11.1%), 1+ in 41 cases (32.5%), 2+ in 43 cases (34.1%), and 3+ in 28 cases (22.2%).

**Table 3:** Condition of immunohistochemical stains and the immunoreactivity

IHC markers	SOX2 (Nu)		SOX2 (Cyto)		CD24		Oct4		IGF-1		FGFR		VEGF		CD44v6	
Vendor	Cell Marque (SP76)				Thermo Scientific		abcam		abcam		abcam		Quartett Immunodiagnostika		Invitrogen	
Dilution	1:100				1:50		1:100		1:100		1:100		1:50		1:200	
Positive control	Normal lung alveoli and squamous cell carcinoma						Testicular seminoma		Normal liver		Normal umbilical cord		Blood vessel		Benign urothelial epithelium	
	No.	(%)	No.	(%)	No.	(%)	No.	(%)	No.	(%)	No.	(%)	No.	(%)	No.	(%)
0	13	(10.3)	66	(52.4)	6	(4.8)	0	(0.0)	13	(10.3)	0	(0.0)	0	(0.0)	14	(11.1)
1+	66	(52.4)	56	(44.4)	79	(62.7)	26	(20.6)	74	(58.7)	7	(5.6)	18	(14.6)	41	(32.5)
2+	45	(35.7)	4	(3.2)	37	(29.4)	73	(57.9)	33	(26.2)	52	(41.3)	78	(63.4)	43	(34.1)
3+	2	(1.6)	0	(0.0)	4	(3.2)	27	(21.4)	6	(4.8)	67	(53.2)	27	(22.0)	28	(22.2)
Total	126	(100)	126	(100)	126	(100)	126	(100)	126	(100)	126	(100)	123	(100)	126	(100)

### Clinicopathological parameters related to tumor recurrence

There was no significant difference between the recurrence and non-recurrence groups based on age, gender, original, revised and combined locations, gross type, ulcer, tumor size, presence or absence of radial resection margin, N stage, and M stage. Although there were no statistically significant relationships between T stage and recurrence, there was a tendency that the recurrence group had a higher T stage than the non-recurrence group. Moreover, there was no statistically significant difference observed between the pathological parameters such as lymphatic invasion, vascular invasion, perineural invasion, histological deferentiation, degree of fibrosis, degree of inflammation, histological subtype and the markers detected by IHC (CK7, CK20, CDX-2, MUC-2, SOX2 (nuclear), SOX2 (cytoplasmic), CD24, Oct4, IGF-1, FGFR1, VEGF, and CD44v6). However, the only significant difference was observed in the original degree of fibrosis (*p* = 0.020) as indicated in Table 4. However, the multivariable regression analysis showed no statistical differences among all clinicopathological parameters according to tumor recurrence (data not shown).

**Table 4:** Clinicopathological parameters related to tumor recurrence

Clinicopathologic parameters	No. (%)		*P* value
	Non-recurrence (*N* = 58)	Recurrence (*N* = 61)	
Age (yrs)	62.4 ± 9.9	64.4 ± 8.9	0.258
Sex			
Male	37 (63.8%)	36 (59.0%)	0.729
Female	21 (36.2%)	25 (41.0%)	
Original location (epicenter)			0.347
Periampullary duodenum	2 (3.4%)	1 (1.6%)	
AOV	20 (34.5%)	16 (26.2%)	
Pancreatic head	15 (25.9%)	16 (26.2%)	
Distal CBD	19 (32.8%)	28 (45.9%)	
Proximal CBD	2 (3.4%)	0 (0.0%)	
Revised location (epicenter)			NA
AOV	0 (0.0%)	0 (0.0%)	
Pancreatic head	20 (34.5%)	14 (23.0%)	
Distal CBD	38 (65.5%)	47 (77.0%)	
Combined location (epicenter)			NA
AOV	0 (0.0%)	0 (0.0%)	
Pancreatic head	17 (34.7%)	13 (26.5%)	
Distal CBD	32 (65.3%)	36 (73.5%)	
Original T stage			0.536
Tis	1 (1.7%)	0 (0.0%)	
T1	13 (22.4%)	8 (13.1%)	
T2	14 (24.1%)	15 (24.6%)	
T3	28 (48.3%)	35 (57.4%)	
T4	2 (3.4%)	3 (4.9%)	
Gross type			NA
Fungating	12 (21.4%)	8 (13.3%)	
Infiltrative	37 (66.1%)	50 (83.3%)	
Ulcerofungating	2 (3.6%)	1 (1.7%)	
Sessile	3 (5.4%)	1 (1.7%)	
Solid	2 (3.6%)	0 (0.0%)	
Ulcer			
Absent	54 (93.1%)	55 (90.2%)	0.805
Present	4 (6.9%)	6 (9.8%)	
Average tumor size	3.0 ± 1.5	3.3 ± 1.7	0.199
Tumor size < 4.5 cm	47 (81.0%)	44 (72.1%)	0.353
≥ 4.5 cm	11 (19.0%)	17 (27.9%)	
Radial resection margin Absent	56 (96.6%)	57 (93.4%)	0.722
Present	2 (3.4%)	4 (6.6%)	
No. of positive lymph nodes	0.7 ± 1.2	1.2 ± 1.8	0.073
No. of dissected lymph nodes	11.6 ± 7.4	11.2 ± 7.0	0.730
N stage			
N0	38 (65.5%)	34 (55.7%)	0.366
N1	20 (34.5%)	27 (44.3%)	
M stage			
M0	55 (94.8%)	60 (98.4%)	0.575
M1	3 (5.2%)	1 (1.6%)	
Lymphatic invasion Absent	38 (65.5%)	30 (49.2%)	0.106
Present	20 (34.5%)	31 (50.8%)	
Vascular invasion			
Absent	51 (87.9%)	53 (86.9%)	1.000
Present	7 (12.1%)	8 (13.1%)	
Perineural invasion Absent	27 (46.6%)	26 (42.6%)	0.805
Present	31 (53.4%)	35 (57.4%)	
Histologic grade			0.845
Well differentiated	17 (29.3%)	17 (27.9%)	
Moderately differentiated	37 (63.8%)	38 (62.3%)	
Poorly differentiated	4 (6.9%)	6 (9.8%)	
Degree of fibrosis			0.020*
None	4 (6.9%)	0 (0.0%)	
Mild	11 (19.0%)	9 (14.8%)	
Moderate	36 (62.1%)	33 (54.1%)	
Severe	7 (12.1%)	19 (31.1%)	
Degree of inflammation			0.415
Mild	21 (36.2%)	28 (45.9%)	
Moderate	30 (51.7%)	29 (47.5%)	
Severe	7 (12.1%)	4 (6.6%)	
Histologic subtype			0.932
Pancreaticobiliary subtype	8 (14.0%)	21 (34.4%)	
Prone to pancreaticobiliary subtype	29 (50.9%)	28 (45.9%)	
Prone to intestinal subtype	10 (17.5%)	8 (13.1%)	
Intestinal subtype	10 (17.5%)	4 (6.6%)	
CK7			
Negative	8 (13.8%)	2 (3.3%)	0.145
1+	5 (8.6%)	5 (8.2%)	
2+	10 (17.2%)	17 (27.9%)	
3+	35 (60.3%)	37 (60.7%)	
CK20			
Negative	28 (48.3%)	42 (68.9%)	0.110
1+	15 (25.9%)	7 (11.5%)	
2+	8 (13.8%)	7 (11.5%)	
3+	7 (12.1%)	5 (8.2%)	
CDX			
Negative	13 (22.4%)	21 (34.4%)	0.086
1+	18 (31.0%)	23 (37.7%)	
2+	10 (17.2%)	10 (16.4%)	
3+	17 (29.3%)	7 (11.5%)	
MUC			
Negative	51 (87.9%)	56 (91.8%)	0.692
Positive	7 (12.1%)	5 (8.2%)	
SOX2 (nuclear) Negative	5 (8.6%)	6 (9.8%)	0.497
1+	31 (53.4%)	31 (50.8%)	
2+	20 (34.5%)	24 (39.3%)	
3+	2 (3.4%)	0 (0.0%)	
SOX2 (cytoplasmic) Negative	30 (51.7%)	32 (52.5%)	0.107
1+	24 (41.4%)	29 (47.5%)	
2+	4 (6.9%)	0 (0.0%)	
CD24			
Negative	4 (6.9%)	2 (3.3%)	0.728
1+	37 (63.8%)	37 (60.7%)	
2+	15 (25.9%)	20 (32.8%)	
3+	2 (3.4%)	2 (3.3%)	
OCT4			
1+	12 (20.7%)	14 (23.0%)	0.943
2+	34 (58.6%)	34 (55.7%)	
3+	12 (20.7%)	13 (21.3%)	
IGF-1			
Negative	7 (12.1%)	6 (9.8%)	0.778
1+	32 (55.2%)	38 (62.3%)	
2+	16 (27.6%)	14 (23.0%)	
3+	3 (5.2%)	3 (4.9%)	
FGFR			
1+	3 (5.2%)	4 (6.6%)	0.697
2+	22 (37.9%)	27 (44.3%)	
3+	33 (56.9%)	30 (49.2%)	
VEGF			
1+	6 (10.3%)	10 (17.2%)	0.547
2+	38 (65.5%)	36 (62.1%)	
3+	14 (24.1%)	12 (20.7%)	
CD44v6 Negative	6 (10.3%)	6 (9.8%)	0.927
1+	18 (31.0%)	22 (36.1%)	
2+	21 (36.2%)	19 (31.1%)	
3+	13 (22.4%)	14 (23.0%)	

### Disease-free survival analysis in recurrence patient

The clinical parameters such as age < 74 (*p* = 0.0221), location of AOV (*p* = 0.014), lower T stage (p = 0.02), size less than 1.5 cm (*p* = 0.0426), lower N stage (N0) (*p* = 0.000391) were significantly associated with better disease-free survival (DFS) in recurrent patients, whereas, no significant correlation was observed in other parameters (Supplementary Fig. 2).

In addition, other pathological parameters including no lymphatic invasion (*p* < 0.0001), histological well differentiation (*p* = 0.00121), intestinal subtype (*p* = 0.0417), and mild fibrosis (*p* = 0.0259) showed a significant association with better DFS (Supplementary Fig. 3). In addition, IHC markers such as CDX-2 (*p* = 0.0245) and FGFR1 (*p* = 0.0181) were also significantly correlated with better DFS (Supplementary Fig. 4). Cox regression analysis showed no significant relation of any clinicopathologic parameters to DFS (data not shown).

### Clinicopathological parameters related to overall survival

**Fig. 1:**
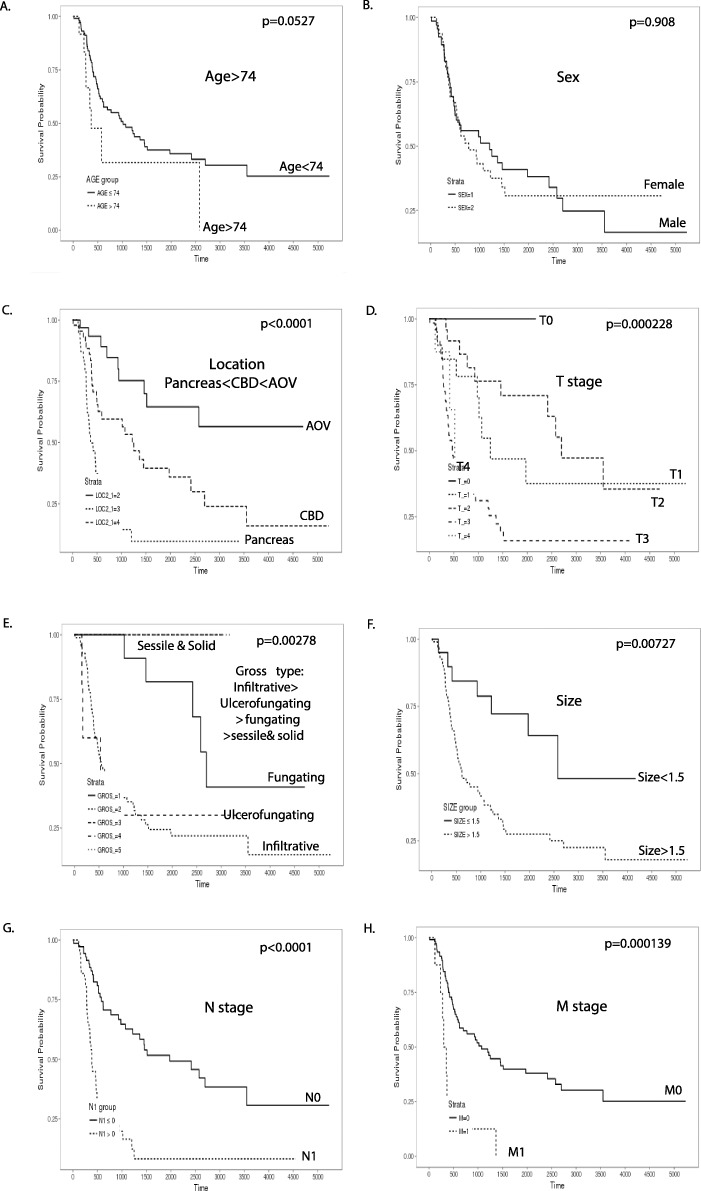
Kaplan-Meier survival analysis on the relationship between overall survival and clinical parameters in periampullary/pancreatic cancers. There was no significant difference according to (**a**) age and (**b**) sex, while there was significant relationship according to (**c**) location, (**d**) T stage, (**e**) gross type, (**f**) size, (**g**) N stage, and (**h**) M stage

**Fig. 2:**
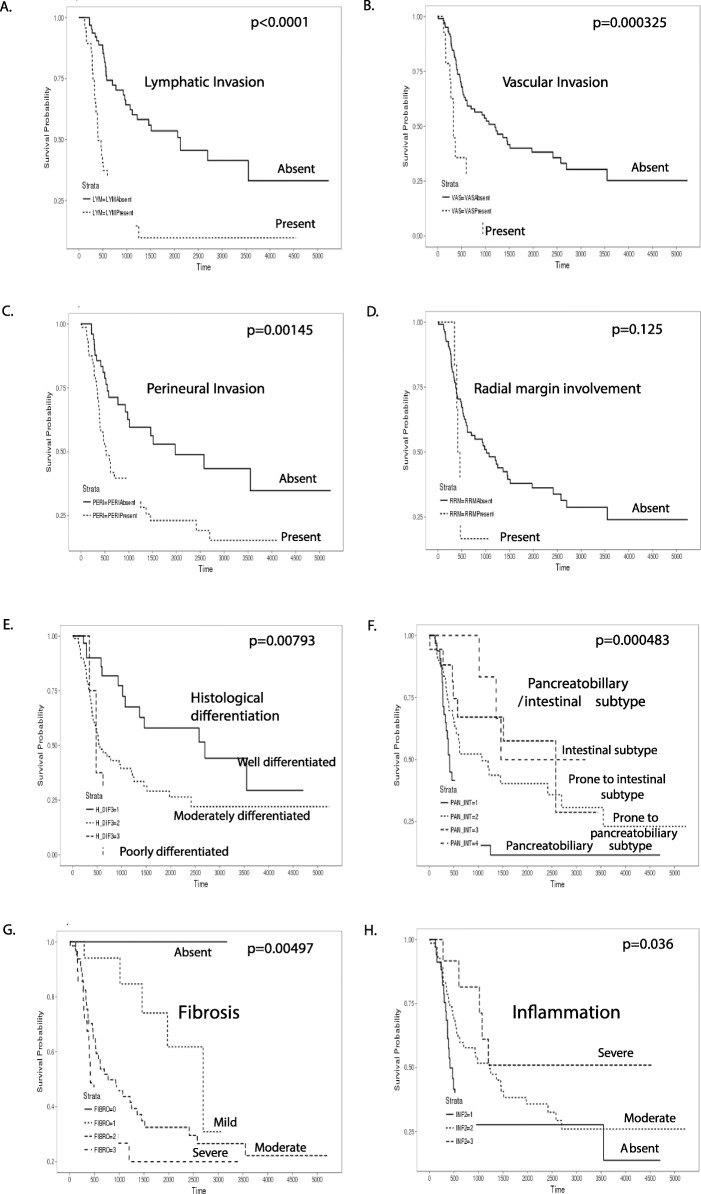
Kaplan-Meier survival analysis on the relationship between overall survival and the pathological parameters in the periampullary/pancreatic cancers. There was significant difference according to **a** lymphatic invasion, **b** vascular Invasion, **c** perineural invasion, **e** histological differentiation, **f** pancreatobillary/intestinal type, **g** fibrosis, and **h** inflammation, whereas there was no significant difference according to **d** radial margin involvement

**Fig. 3:**
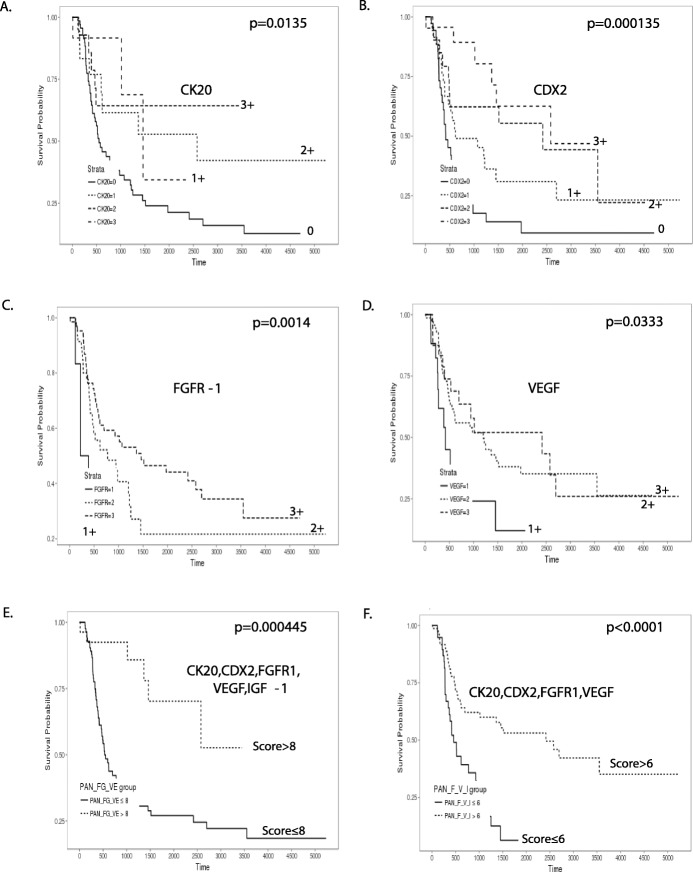
Kaplan-Meier overall survival analysis on the relationship between overall survival and expression level of the IHC markers in periampullary/pancreatic cancers. There was significant difference according to **a** CK20, **b** CDX2, **c** FGFR1, **d** VEGF, **e** combined CK20, CDX2, FGFR1, VEGF, and IGF-1 (combined score of 5 marker expression levels, 2¬15), and **f** combined CK20,CDX2, FGFR1, and VEGF (combined score of 4 marker expression levels, 2¬12)

Among clinical parameters, age (*p* = 0.0527) and gender (*p* = 0.908) were not associated with overall survival. On the other hand, location of AOV (*p* < 0.0001), lower T stage (*p* = 0.000228), sessile and solid gross type (*p* = 0.00278), size less than 1.5 cm (*p* = 0.00727), lower N stage (N0) (p < 0.0001), and lower M stage (M0) (*p* = 0.000139) were significantly related to better overall survival (Fig. 1). Among pathological parameters, better overall survival was related to no lymphatic invasion (*P* < 0.0001), no vascular invasion (*p* = 0.000325), no perineural invasion (*p* = 0.00145), histological well differentiation (*p* = 0.000793), intestinal subtype (*p* = 0.000483), no fibrosis (*p* = 0.00497), and severe inflammation (*p* = 0.036) (Fig. 2). In addition, expression of four IHC markers, higher expression of intestinal-type markers, CK20 (*p* = 0.0135) and CDX2 (*p* = 0.000135), and higher expression of EMT markers, FGFR1 (p = 0.0014) and VEGF (*p* = 0.0333) were significantly related to better overall survival (Fig. 3). The combined panel expression score more than 8 of CK20, CDX2, FGFR1, VEGF, and IGF-1 was significantly related to better overall survival (*p* = 0.000445) as well as the combined panel expression score more than 6 of CK20, CDX2, FGFR1, and VEGF (*p* < 0.0001) (Fig. 3). Cox regression analysis also showed a significant relationship of N stage, lymphatic invasion, degree of inflammation, pancreaticobiliary/intestinal subtypes, expression of intestinal markers, CK20 and CDX2, and EMT markers, FGFR1 and VEGF (Supplementary Table 2, Supplementary Fig. 5).

## Discussion

The two major findings in this study are, first, FGFR1 could be a promising prognostic marker for periampullary cancers, and second, peritumoral fibrosis was associated with tumor recurrence in periampullary cancer patients. We also confirmed significant relationship between overall survival and previously known clinicopathological prognostic markers, such as age, location, T stage, gross type, size, N stage, M stage, lymphatic invasion, vascular invasion, perineural invasion, histological differentiation, inflammation and the staining pattern of the IHC markers (CK20, CDX2), as described in the literature [1, 12].

In the present study, FGFR1 is significantly associated with overall survival and disease-free survival in periampullary/pancreatic cancer patients. FGFR1 has been known to be related to the prognosis of several human cancers [9, 13]. It is a member of the tyrosine kinase family and shares similar structural morphology with VEGFRs and platelet-derived growth factor receptors (PDGFRs), which implies the potential role of FGFR1 in the carcinogenesis of many human cancers [9, 13]. Many reports including data from the cancer genome atlas study have provided evidence regarding the involvement of FGFRs in the carcinogenesis of several cancers such as primary lobular breast carcinomas (20%) [14], lung cancer (22%) [15], and pancreatic cancer (5%) [16]. However, the prognostic impact of FGFR1 expression in cancer shows conflicting results depending on cancer types. Higher expression of the FGFR1 gene predicts poor overall survival and shorter disease-free survival in esophageal squamous cell carcinoma [17] and similar results were observed in non-small-cell lung cancer, particularly squamous cell carcinoma [18].

On the other hand, a recent study performed in the Korean patients showed that FGFR1 positive pancreatic cancer had better overall survival as compared to FGFR1 negative pancreatic cancer [19], which is consistent with the findings of our study. Although the precise reason for this discrepancy in different cancer types is unclear, it could probably be due to the different pathogenic roles of FGFR1 in various cancers. High expression of FGFR1 might cause a severe desmoplastic reaction (increased fibrosis) and could be protective or antitumorigenic in pancreatic cancers.

On the other hand, in this study, we found that the degree of peritumoral fibrosis was related to tumor recurrence. Although this finding seems conflicting with the first finding, high FGFR1 expression is correlated with the degree of fibrosis but is not strictly linked together. In pancreatic cancer, pancreatic stellate cells (PSCs) in the stroma are considered as the sprouting seeds of cancer progression and metastasis [20]. These are essential components of the tumor-stromal organization and are usually present as quiescent or inactive cells in normal pancreatic tissue. These cells are believed to play a key role in extracellular matrix production and regulate or promote EMT [2, 20]. It is possible that after surgery, tumors with a higher degree of fibrosis might have increased remnant fibrotic tissue containing activated PSCs, and this could be the foci of recurrence. In a recent study in colorectal cancers, fibrosis in metastatic lymph nodes was strongly associated with poor overall survival and relapse-free survival [21], suggesting that the fibrosis in the metastatic lymph nodes can be a potential foci of tumor recurrence. In our study, recurrence was also related to lymph node metastasis.

EMT is very important for the progression and metastasis of many cancers. It is particularly crucial in pancreatic cancer because histologically, pancreatic cancer is characterized by increased mesenchymal as well as epithelial features compared to other adenocarcinomas in other organs. In EMT, the epithelial cells lose polarity and cell to cell contact, have decreased E-cadherin expression and increased vimentin expression during the mesenchymal transition [8]. However, the loss of E-cadherin is usually exclusively found in metastatic lesions and the vimentin is well known for non-specific, frequent coexpression in pancreatic cancers. Therefore, the feasibility as a prognostic marker seems to be limited although a few studies have suggested these as potential prognostic markers [22].

On the other hand, the EMT marker VEGF shares similar structural morphology with FGFRs, and both are very important for angiogenesis [9]. It has been reported that the expression of VEGF and FGFR1 were correlated with microvessels density [13]. Generally, VEGF is associated with poor overall survival in cancer. Yousuke et al. demonstrated that high VEGF expression showed significantly worse survival compared to low VEGF expression in pancreatic adenocarcinoma [23]. Surprisingly, in our study, we found a positive relationship between VEGF expression and overall survival, which is opposed to that of previous studies. In addition, the combined panel expression of CK20, CDX2, FGFR1, VEGF, and IGF-1 and CK20, CDX2, FGFR1, and VEGF were also useful prognostic markers for better survival.

In terms of stem cell markers, no stems cell markers including Oct4, SOX2, CD24, and CD44v6, showed significant relationship either with overall survival and disease-free survival. Higher expression of SOX2 and Oct4 has been reported to be associated with worse survival in some cancers, such as esophageal carcinoma [24]. As mentioned in the introduction, the aberrant expression of stem cell markers, such as Oct4, SOX2, CD24, CD44, PDX-1, SHH, and CD133, was suggested to be related to the prognosis in pancreatic cell lines, according to several studies [7, 25]. However, there are no studies so far conducted on the human hosts but only in the cell lines about the aforementioned markers. Our study investigated the clinical and prognostic significance of the stem cell marker expression in a large number of pancreatic cancer samples, and the result was not significant among all markers, which was opposed to the results of the studies using the cell lines. One thing to notice, Quint et al. have reported that the expression of stem cell markers, Ptc and PDX-1, is related to survival in certain grades of pancreatic cancers using 51 cases [26].

In this study, we reevaluated the T stage according to the tumor epicenter and, 22 out of 126 cases (17.4%) have been revised. Although it is not as high as reported by Adsay et al. (39%) [2], the number of cases with the revised T stage was considerably high and therefore, cannot be neglected. Due to the anatomic complexity, determining the tumor epicenter correctly when the tumor is detected in the advanced stage is a complicated issue. In this study, although we evaluated the difference in T staging based on the 7th AJCC cancer staging system, the results would be similar in the 8th edition as well. The determination of tumor epicenter is the most important factor to differentiate AOV, pancreatic, and CBD cancers and the T stage of 8th edition AJCC system is also based on the different size standards according to the location [27]. In our suggestion, pancreaticobiliary/intestinal subtypes, according to the expression status of CK20 and CDX2, can be a good supportive parameter for better TNM staging. The combined panel markers including CK20, CDX2, FGFR1, VEGF, and IGF-1 or CK20, CDX2, FGFR1, and VEGF, also can be good prognostic markers.

## Conclusions

In this study, we identified fibrosis and FGFR1 expression as new prognostic markers to predict tumor recurrence and overall survival, respectively. Moreover, there was no significant correlation between stem cell markers and overall survival. Further basic studies on the VEGF and FGFR1 expression and the relationship between the markers need to be investigated in relation to EMT of PACs.

## Supplementary information

**Additional file 1: Table S1.** Comparison of T stage in AJCC cancer staging system 7th edition (2010) according to the tumor location. **Table S2.** Cox regression analysis for overall survival of periampullary/pancreatic cancer patients.

**Additional file 2: Figure S1.** Representative images of the immunohistochemical stainings using the tissue microarray of periampullary/ periampullary cancers (× 400). (a) Sox2 cytoplasm and nucleus (b) CD24 (c) OCT4 (d) IGF-1 (e) FGFR1, (f) VEGF (g) CD44v6 (h) CK7 (i) CK20 (j) CDX-2 (k) MUC2, with negative (no expression), 1+ (low expression), 2+ (moderate expression) and 3+ (high expression) intensity. **Figure S2.** Kaplan-Meier survival analysis on the relationship between disease free survival and clinical parameter in recurrent periampullary/pancreatic cancers . There was significant difference according to (A) age, (C) location, (D) T stage, (F) Size, and (G) N stage. While there was no significant difference according to (B) sex, (E) gross type, and (H) M stage. **Figure S3.** Kaplan-Meier survival analysis on the relationship between disease free survival and pathological parameter in recurrent periampullary/pancreatic cancers . There was significant difference according to (A) lymphatic invasion, (E) histological differentiation, (F) pancreatobillary type vs intestinal type, and (G) fibrosis, while there was no significant relationship according to (B) vascular Invasion, (C) perineural invasion, (D) radial margin involvement, and (H) inflammation. **Figure S4.** Kaplan-Meier survival analysis on the relationship between disease free survival analysis and expression level of the IHC markers in recurrent periampullary/pancreatic cancers. There was no significant difference according to (A) CK20, and (D) VEGF, while there was significant difference according to (B) CDX2, and (C) FGFR1. **Figure S5.** Cox regression analysis of FGFR in periampullary/pancreatic cancer patients. (A) Survival probability of FGFR1+, 2+, 3+ groups, (B) Log-Log survival probability of FGFRs 1+, 2+, 3+ groups.

## Data Availability

The original dataset of this study is available at http://www.researchgate.net/profile/Yosep_Chong.

## Abbreviations

AJCC: American Joint Cancer Committee
CT: Computed tomography
EMT: Epithelial-mesenchymal transition
FGFR1: Fibroblast growth factor receptor 1
IGF-1: Insulin-like growth factor-1
OCT-4: Octamer transcription factor-4
PET: Position emission tomography
PSC: Pancreatic stellate cells
TNM: Tumor, node, metastases
UICC: Union International Contre le Cancer staging system
VEGF: Vascular Endothelial growth factor
