# Supplementary material for: Prognostic significance of stem cell/ epithelial-mesenchymal transition markers in periampullary/pancreatic cancers: FGFR1 is a promising prognostic marker
Source: BMC Cancer. 2020 Mar 14;20:216. doi: 10.1186/s12885-020-6673-2 (PMC7071628; doi:10.1186/s12885-020-6673-2)
Supplement: Supplementary file 1 — Additional file 1: Table S1. Comparison of T stage in AJCC cancer staging system 7th edition (2010) according to the tumor location. Table S2. Cox regression analysis for overall survival of periampullary/pancreatic cancer patients. [file 12885_2020_6673_MOESM1_ESM.docx]

**Supplementary Table 1. Comparison of T stage in AJCC cancer staging system 7th edition (2010) according to the** **tumor location**

| **Distal bile duct** | |
| --- | --- |
| T1 | Confined to the bile duct (histologically) |
| T2 | Invading beyond the wall of the bile duct |
| T3 | Invading the gallbladder, pancreas, **duodenum**, or other adjacent organs |
| T4 | Invading the celiac axis, or the superior mesenteric artery |
| **Ampulla of Vater (AOV)** | |
| T1 | Limited to AOV |
| T2 | Invading the duodenal wall |
| T3 | Invading the pancreas |
| T4 | Extending the peripancreatic soft tissue or another adjacent organ |
| **Pancreas** | |
| T1 | Limited to the pancreas, < 2cm |
| T2 | Limited to the pancreas, > 2cm |
| T3 | Extending beyond the pancreas |
| T4 | Invading the celiac axis or the superior mesenteric artery |
| **Duodenum** | |
| T1 | Invading submucosal |
| T2 | Invading proper muscle |
| T3 | Invading subserosal or perimuscular tissue with extension less than 2 cm |
| T4 | Perforating visceral peritoneum or direct invading to the other organ (pancreas or bile duct) |

**Supplementary Table 2. Cox regression analysis for overall survival of periampullary/pancreatic cancer patients**

| **Variables** | | **P value** | **OR** | **95.0% CI for OR** | |
| --- | --- | --- | --- | --- | --- |
| Location | AOV | 0.254 |  |  | |
|  | Pancreas | 0.201 | 3.677 | | 0.499 - 27.069 |
|  | CBD | 0.107 | 5.740 | | 0.684 - 48.137 |
| T stage | Tis | 0.002 |  | |  |
|  | T1 | **0.031*** | 0.044 | | 0.003 - 0.758 |
|  | T2 | **0.048*** | 6.404 | | 1.020-40.193 |
|  | T3 | 0.098 | 0.052 | | 0.002-1.719 |
| Gross type | Fungating | 0.062 |  | |  |
|  | Ulcerofungating | **0.047*** | 0.032 | | 0.001-0.960 |
|  | Infiltrative | 0.996 | 0.000 | | 0.000 |
|  | Sessile | 0.356 | 6.389 | | 0.124-328.774 |
|  | Solid | 0.995 | 0.000 | | 0.000 |
| Size <4.5cm | <4.5 vs. >=4.5cm | 0.460 | 0.616 | | 0.170-2.227 |
| Resected margin  involvement | Absent vs. Present | 0.469 | 0.379 | | 0.028-5.221 |
| N stage | N0 vs. N1 | **0.012*** | 26.689 | | 2.063-345.343 |
| M stage | M0 vs. M1 | 0.641 | 0.597 | | 0.068-5.210 |
| Lymphatic invasion | Absent vs. Present | **0.026** | 0.052 | | 0.004-0.707 |
| Perineural invasion | Absent vs. Present | 0.375 | 0.391 | | 0.049-3.104 |
| Vascular invasion | Absent vs. Present | 0.615 | 1.551 | | 0.280-8.593 |
| Histologic grade | Well  Moderate | 0.156  0.484 | 0.444 | | 0.046-43.19 |
|  | Poor | 0.210 | 10.317 | | 0.269-395.365 |
| Histologic subtype | Pancreaticobiliary subtype | 0.001 |  | |  |
|  | Prone to pancreaticobilary subtype | **0.000*** | 0.000 | | 0.000-0.009 |
|  | Prone to intestinal subtype | **0.001*** | 0.000 | | 0.000-0.038 |
|  | Intestinal subtype | **0.003*** | 0.000 | | 0.000-0.48 |
| Degree of fibrosis | Mild  Moderate  Severe | 0.616  0.338  0.330 | 3.839  4.174 | | 0.245-60.208  0.236-73.955 |
| Degree of inflammation | Mild | 0.000 |  | |  |
|  | Moderate | **0.000*** | 0.046 | | 0.010-0.204 |
|  | Severe | **0.002*** | 0.011 | | 0.001-0.179 |
| CK20 | Negetive | 0.000 |  | |  |
|  | 1+ | **0.000*** | 172.063 | | 12.362-2394.981 |
|  | 2+ | 0.131 | 7.352 | | 0.533-97.705 |
|  | 3+ | **0.000*** | 6305.322 | | 124.906-318297.044 |
| CDX2 | Negetive | 0.000 |  | |  |
|  | 1+ | **0.000*** | 6941.803 | | 85.029-566731.729 |
|  | 2+ | **0.002*** | 1411.220 | | 14.809-134478.015 |
|  | 3+ | **0.462*** | 4.532 | | 0,081-253.449 |
| VEGF | 1+ | 0.119 |  | |  |
|  | 2+ | **0.039*** | 4.101 | | 1.070-15.711 |
|  | 3+ | 0.241 | 3.821 | | 0.406-35.941 |
| FGFR | 1+  2+  3+ | 0.072  0.192  **0.040*** | 0.272  0.061 | | 0.022-2.153  0.004-0.879 |
