# Supplementary material for: Prognostic significance of stem cell/ epithelial-mesenchymal transition markers in periampullary/pancreatic cancers: FGFR1 is a promising prognostic marker
Source: BMC Cancer. 2020 Mar 14;20:216. doi: 10.1186/s12885-020-6673-2 (PMC7071628; doi:10.1186/s12885-020-6673-2)
Supplement: Supplementary file 2 — Additional file 2: Figure S1. Representative images of the immunohistochemical stainings using the tissue microarray of periampullary/ periampullary cancers (× 400). (a) Sox2 cytoplasm and nucleus (b) CD24 (c) OCT4 (d) IGF-1 (e) FGFR1, (f) VEGF (g) CD44v6 (h) CK7 (i) CK20 (j) CDX-2 (k) MUC2, with negative (no expression), 1+ (low expression), 2+ (moderate expression) and 3+ (high expression) intensity. Figure S2. Kaplan-Meier survival analysis on the relationship between disease free survival and clinical parameter in recurrent periampullary/pancreatic cancers . There was significant difference according to (A) age, (C) location, (D) T stage, (F) Size, and (G) N stage. While there was no significant difference according to (B) sex, (E) gross type, and (H) M stage. Figure S3. Kaplan-Meier survival analysis on the relationship between disease free survival and pathological parameter in recurrent periampullary/pancreatic cancers . There was significant difference according to (A) lymphatic invasion, (E) histological differentiation, (F) pancreatobillary type vs intestinal type, and (G) fibrosis, while there was no significant relationship according to (B) vascular Invasion, (C) perineural invasion, (D) radial margin involvement, and (H) inflammation. Figure S4. Kaplan-Meier survival analysis on the relationship between disease free survival analysis and expression level of the IHC markers in recurrent periampullary/pancreatic cancers. There was no significant difference according to (A) CK20, and (D) VEGF, while there was significant difference according to (B) CDX2, and (C) FGFR1. Figure S5. Cox regression analysis of FGFR in periampullary/pancreatic cancer patients. (A) Survival probability of FGFR1+, 2+, 3+ groups, (B) Log-Log survival probability of FGFRs 1+, 2+, 3+ groups. [file 12885_2020_6673_MOESM2_ESM.pptx]

## Slide 1
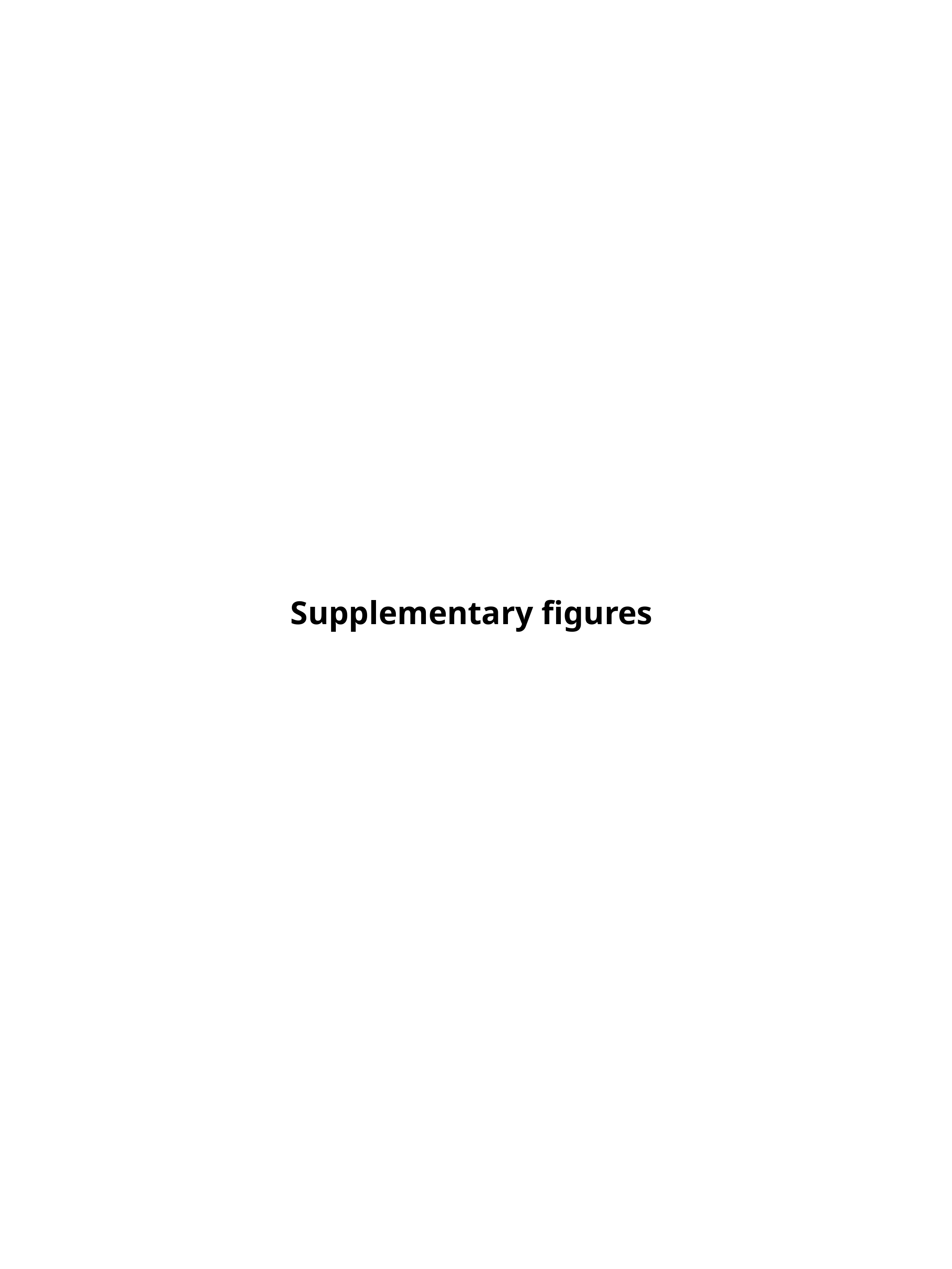

Supplementary figures

## Slide 2
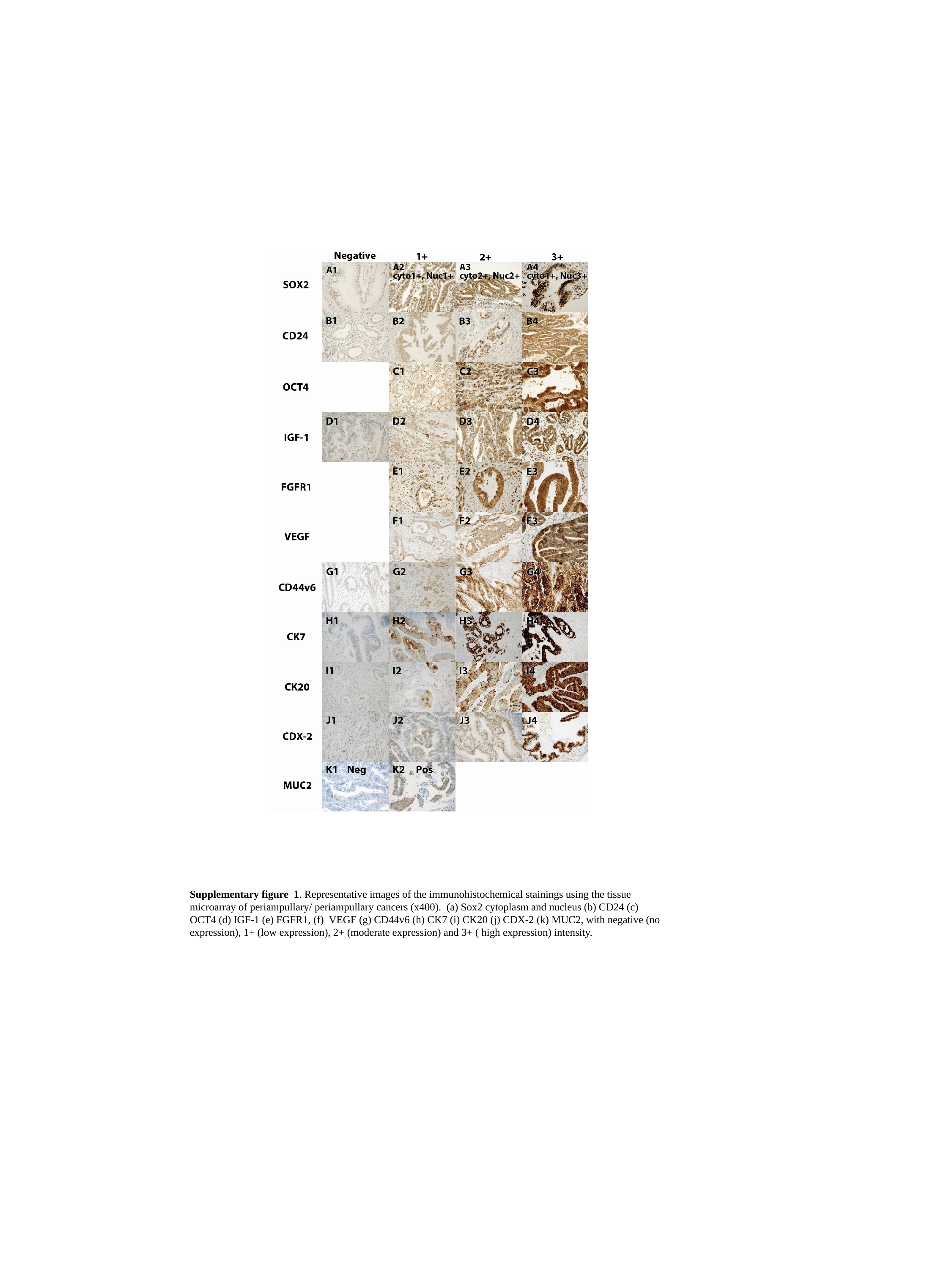

Supplementary figure 1. Representative images of the immunohistochemical stainings using the tissue microarray of periampullary/ periampullary cancers (x400). (a) Sox2 cytoplasm and nucleus (b) CD24 (c) OCT4 (d) IGF-1 (e) FGFR1, (f) VEGF (g) CD44v6 (h) CK7 (i) CK20 (j) CDX-2 (k) MUC2, with negative (no expression), 1+ (low expression), 2+ (moderate expression) and 3+ ( high expression) intensity.

## Slide 3
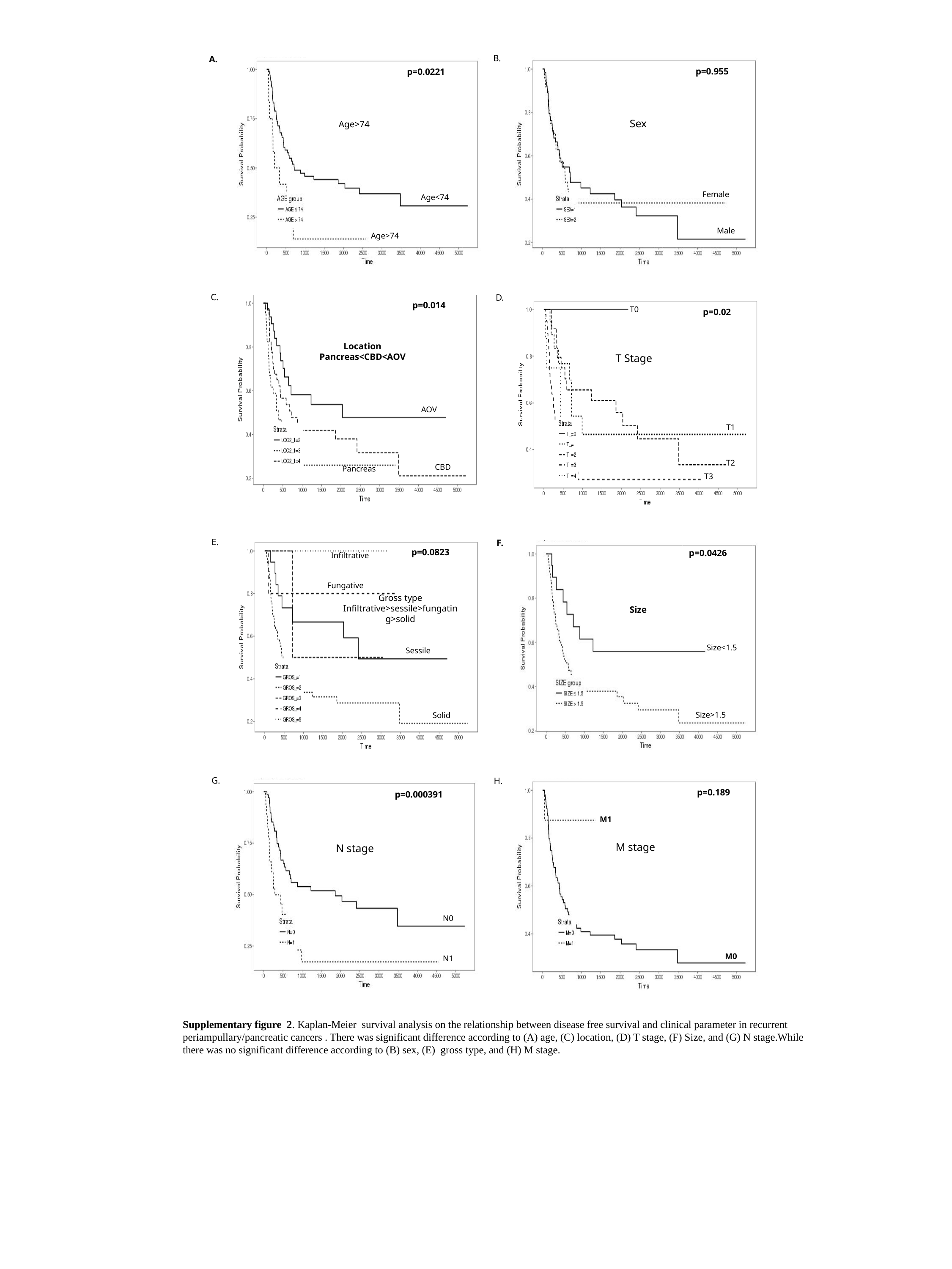

B.
A.
p=0.0221
Age>74
Sex
p=0.955
C.
D.
p=0.014
Location
Pancreas<CBD<AOV
T Stage
p=0.02
E.
F.
Gross type Infiltrative>sessile>fungating>solid
p=0.0823
Size
p=0.0426
G.
H.
N stage
p=0.000391
M stage
p=0.189
Female
Age<74
Male
Age>74
T0
AOV
T1
T2
CBD
Pancreas
T3
Infiltrative
Fungative
Size<1.5
Sessile
Size>1.5
Solid
M1
N0
M0
N1
Supplementary figure 2. Kaplan-Meier survival analysis on the relationship between disease free survival and clinical parameter in recurrent periampullary/pancreatic cancers . There was significant difference according to (A) age, (C) location, (D) T stage, (F) Size, and (G) N stage.While there was no significant difference according to (B) sex, (E) gross type, and (H) M stage.

## Slide 4
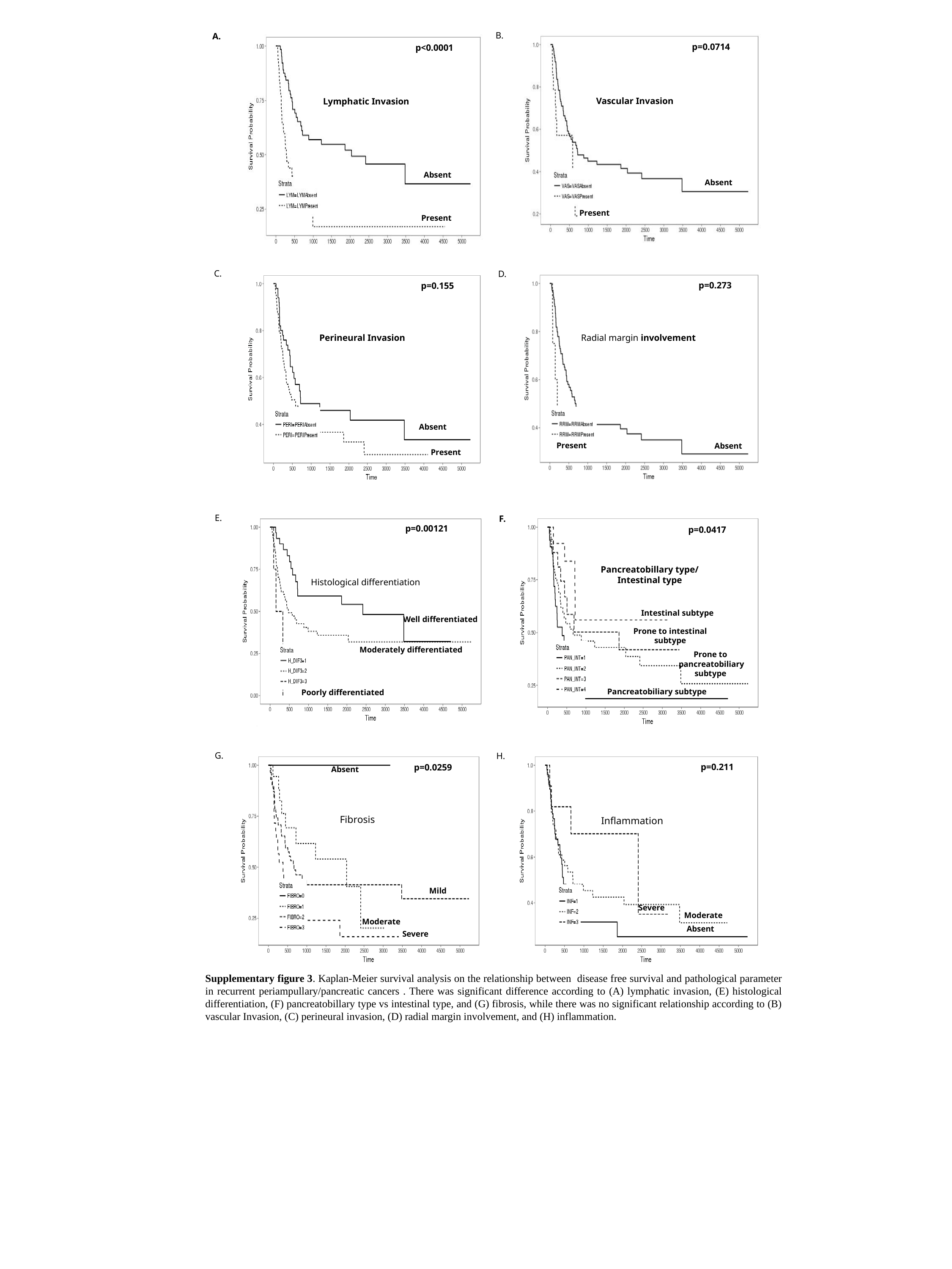

B.
A.
Lymphatic Invasion
p<0.0001
Vascular Invasion
p=0.0714
C.
D.
Radial margin involvement
p=0.273
Perineural Invasion
p=0.155
E.
F.
Pancreatobillary type/ Intestinal type
p=0.0417
Histological differentiation
p=0.00121
G.
H.
Fibrosis
p=0.0259
Inflammation
p=0.211
Absent
Absent
Present
Present
Absent
Present
Absent
Present
Intestinal subtype
Well differentiated
Prone to intestinal subtype
Moderately differentiated
Prone to pancreatobiliary subtype
Pancreatobiliary subtype
Poorly differentiated
Absent
Mild
Severe
Moderate
Moderate
Absent
Severe
Supplementary figure 3. Kaplan-Meier survival analysis on the relationship between disease free survival and pathological parameter in recurrent periampullary/pancreatic cancers . There was significant difference according to (A) lymphatic invasion, (E) histological differentiation, (F) pancreatobillary type vs intestinal type, and (G) fibrosis, while there was no significant relationship according to (B) vascular Invasion, (C) perineural invasion, (D) radial margin involvement, and (H) inflammation.

## Slide 5
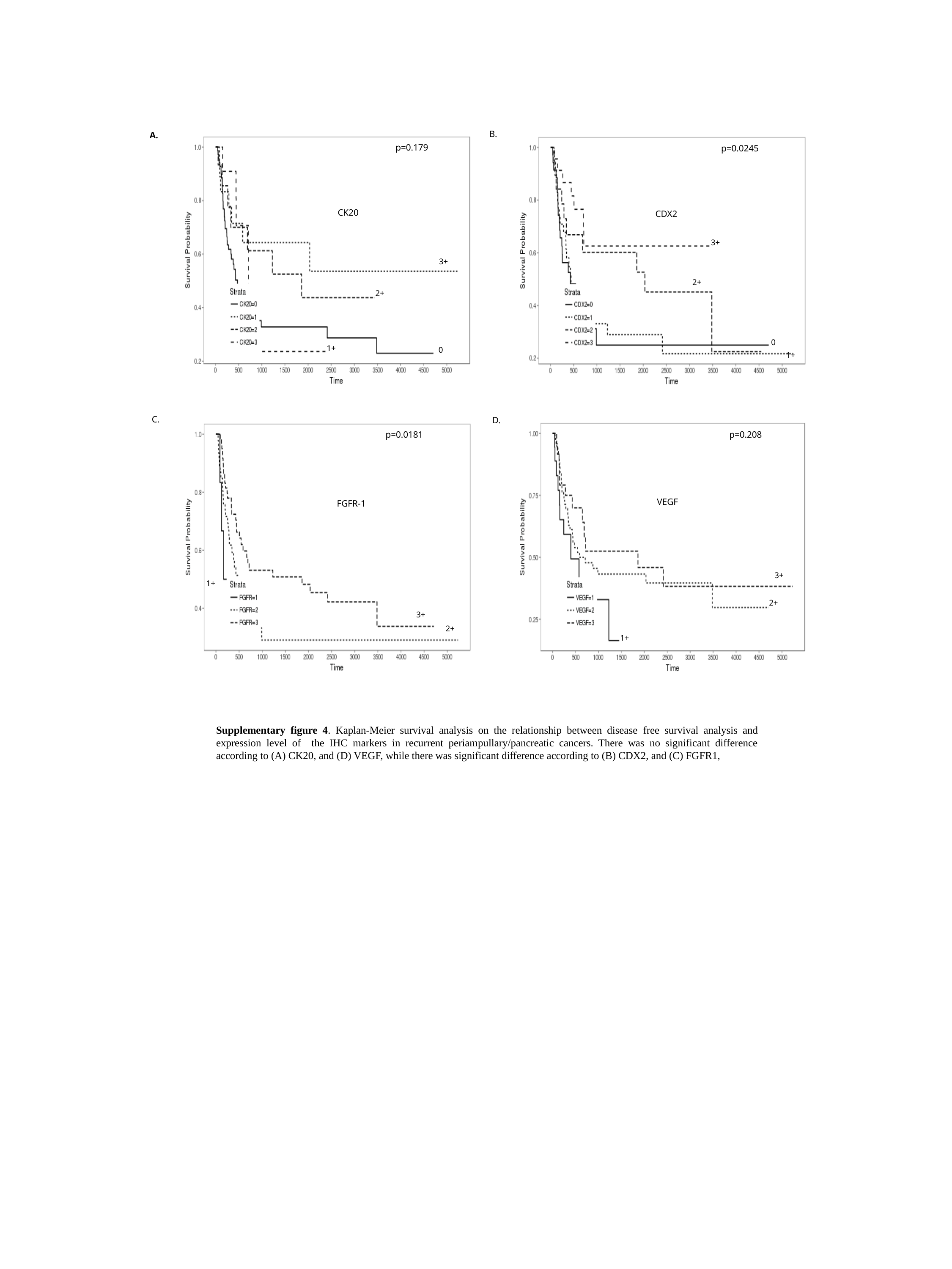

B.
A.
CDX2
p=0.0245
CK20
p=0.179
C.
D.
VEGF
p=0.208
FGFR-1
p=0.0181
3+
3+
2+
2+
0
1+
0
1+
3+
1+
2+
3+
2+
1+
Supplementary figure 4. Kaplan-Meier survival analysis on the relationship between disease free survival analysis and expression level of the IHC markers in recurrent periampullary/pancreatic cancers. There was no significant difference according to (A) CK20, and (D) VEGF, while there was significant difference according to (B) CDX2, and (C) FGFR1,

## Slide 6
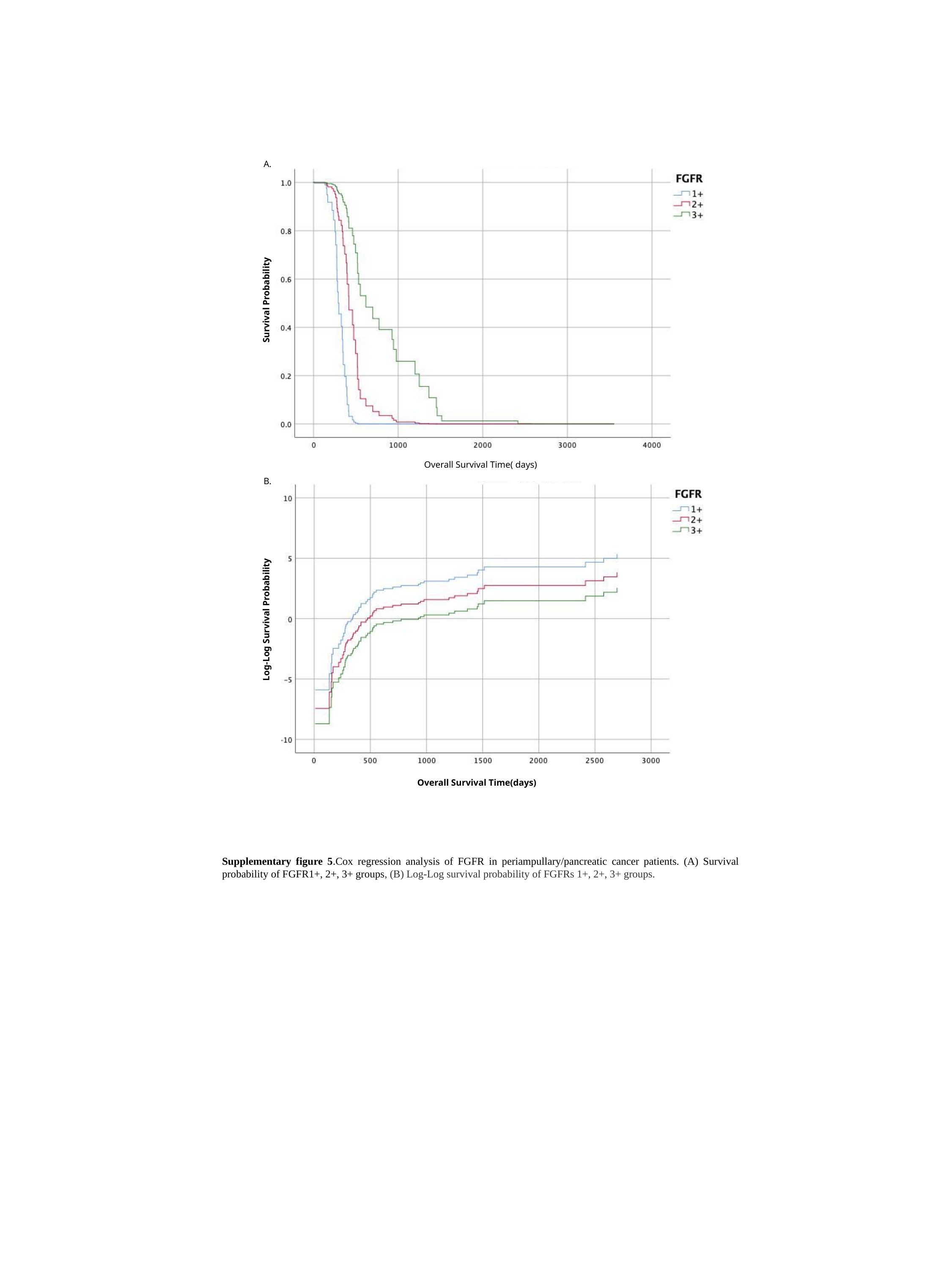

A.
 Survival Probability
Overall Survival Time( days)
B.
Overall Survival Time(days)
Log-Log Survival Probability
Supplementary figure 5.Cox regression analysis of FGFR in periampullary/pancreatic cancer patients. (A) Survival probability of FGFR1+, 2+, 3+ groups, (B) Log-Log survival probability of FGFRs 1+, 2+, 3+ groups.
